# The impact of trait anxiety on academic procrastination among college students: the mediating role of academic self-efficacy and the moderating role of autonomous learning motivation

**DOI:** 10.3389/fpsyg.2026.1770357

**Published:** 2026-04-01

**Authors:** Junqiang Fan, Liting Bu, Tang Ming, Jingbo Shan, Yuxin Huang, Jinquan Sun

**Affiliations:** 1 School of Management, Zhejiang University of Science and Technology, Hangzhou, Zhejiang, China; 2 Anji Campus, Zhejiang University of Science and Technology, Huzhou, Zhejiang, China; 3 The First School of Clinical Medicine, Zhejiang Chinese Medical University, Hangzhou, Zhejiang, China

**Keywords:** academic procrastination, academic self-efficacy, autonomous learning motivation, mediating effects, trait anxiety

## Abstract

**Introduction:**

This study aimed to elucidate the mechanisms through which trait anxiety influences academic procrastination among college students, with a focus on the roles of academic self-efficacy and autonomous learning motivation.

**Methods:**

A survey was conducted with 3,370 college students. Correlation, mediation, and moderated mediation analyses were employed to examine the interrelationships among trait anxiety, academic self-efficacy, autonomous learning motivation, and academic procrastination.

**Results:**

Over 50% of participants reported moderate or higher levels of academic procrastination (M = 23.18, SD = 5.33). Trait anxiety showed significant positive correlations with academic procrastination, while academic self-efficacy and autonomous learning motivation were negatively correlated with both trait anxiety and procrastination. Trait anxiety significantly predicted academic procrastination (*β* = 0.284–0.401, 95% CI). Academic self-efficacy partially mediated this relationship (indirect effect *β* = 0.123–0.215, 95% CI). Furthermore, autonomous learning motivation moderated the link between trait anxiety and academic self-efficacy, such that the negative impact of trait anxiety on self-efficacy and consequently on procrastination was stronger for students with lower autonomous motivation.

**Discussion:**

The findings suggest that trait anxiety contributes to academic procrastination both directly and indirectly through diminished academic self-efficacy. Autonomous learning motivation serves as a protective buffer. Interventions aimed at reducing academic procrastination should address students’ anxiety, enhance their academic self-efficacy, and foster autonomous learning motivation.

## 1 Introduction

Currently, the prevalent phenomenon of academic procrastination in tertiary education cohorts underscores a pervasive concern in contemporary scholarly discourse. According to empirical research findings, approximately 54% of undergraduate students in China exhibit moderate or higher levels of academic procrastination, while prevalence rates ranging from 80 to 95% have been reported in the United States (Zeng and Li, 2019; Steel, 2007). Academic procrastination is deliberate postponement in starting or finishing coursework (Li et al., 2021; Goroshit, 2018). This behavior extends its impact beyond academic performance to detrimentally affect students’ holistic health dimensions encompassing physiological stability, psychological equilibrium, and affective states, and overall life satisfaction (Liu et al., 2023; Peixoto et al., 2021). Therefore, it is an important task in university student affairs to explore the mechanisms underlying academic procrastination and to implement proactive intervention and prevention strategies to reduce such behaviors at their source.

Research as revealed multiple factors of the psychological mechanisms of academic procrastination. The conceptual model of procrastination (Procee et al., 2013) proposes that negative emotions are key factors of procrastination behavior (Song et al., 2015). At the same time, procrastination can lead to emotional problems such as depression and anxiety. Individuals with high levels of anxiety are more likely to procrastinate when facing tasks. However, procrastination does not effectively relieve anxiety. Instead, it may intensify anxiety (Gadosey et al., 2021), which in turn leads to more severe procrastination (Shan H. et al., 2016), thereby forming a vicious cycle. The relationship between procrastination and anxiety among undergraduates is complex and dynamic, where anxiety serves as an antecedent variable of procrastination and the two influence each other reciprocally (Wu et al., 2025). Strategies such as self-monitoring, self-reward, and time management may help individuals improve procrastination behavior and break the procrastination cycle.

Academic procrastination has a multidimensional structure, which is examined by scholars’ structural or multivariate models. It is influenced by both internal and external factors, including personality, motivation, parenting style, and characteristics of academic tasks, and resulting from the interaction of behavioral, cognitive, and emotional elements (Li and Lü, 2022; Wang and Gao, 2021). A structural equation model (Özbay et al., 2025) demonstrates that nomophobia, netlessphobia, academic self-efficacy, and attentional control jointly predicted academic procrastination, explaining a substantial proportion of its variance. Karagöz and Özbay (2025) identified academic procrastination as a mediating variable linking digital-related anxieties, thereby highlighting its explanatory role within broader psychological systems. These findings reveal indicate that academic procrastination not only is conceptualized solely as a dependent outcome but also as a dynamic mechanism embedded within complex interactional frameworks. According to ecological systems theory (Ji, 2016), academic procrastination is the result of interactions between individuals and their environment (Li, 2021). In the Internet era, the online environment has a substantial impact on individuals, particularly posing threats to adolescents’ learning status and mental health (Li et al., 2023). Internet addiction is a by-product of the information age, characterized by excessive or pathological Internet use. To some extent, it replaces the sense of achievement and satisfaction derived from completing academic tasks on time. Xue (2024) found that adolescent Internet addiction significantly and positively predicted academic procrastination, with self-control and anxiety serving as multiple mediators in this relationship. Additionally, academic procrastination directly influenced mobile phone dependence among college students, and also affected it through independent pathways involving negative cognitive emotion regulation strategies and anxiety (Yang et al., 2025). According to motivational factor theory (Conti, 2000), motivational components also are key drivers of academic procrastination behavior, and academic procrastination is negatively correlated with learners’ self-efficacy. Empirical research has shown that academic procrastination is associated with both self-efficacy and autonomous academic motivation (Steel et al., 2022). However, the specific mechanisms underlying these relationships remain unclear.

Based on the above literature, the present study constructs a moderated mediation model to explain the effect of trait anxiety on academic procrastination among college students. It further examines the potential mechanisms of academic self-efficacy and autonomous learning motivation. This study aims to provide a theoretical basis for educators and mental health practitioners, helping students better regulate their emotions and behaviors when facing academic stress, and thereby improve academic performance and psychological well-being.

### 1.1 The association of anxiety with academic procrastination

Speilberger et al. (1983) categorized anxiety into trait anxiety and state anxiety, with this typology grounded in temporal persistence characteristics. Trait anxiety represents an individual’s inherent tendency toward anxiety as a stable dispositional characteristic exhibiting inter-individual variability. State anxiety denotes a transient anxiety state induced by situational factors, manifesting immediately with a defined magnitude (Geng et al., 2019). Those with elevated trait anxiety easily feel threatened, which in turn triggers a high level of anxiety (He, 2019). Procrastination demonstrates strong linkages to negative emotions, and researchers are increasingly focusing on the connection between anxiety and academic procrastination. Procrastination represents a failure in emotional regulation—a behavior where individuals forego long-term positive outcomes to cope with negative emotions, as posited by the Short-term Mood Regulation Theory (Sirois and Pychyl, 2013). Plenty of studies have consistently proved the significance of anxiety as a pivotal predictor of procrastination behavior (Li et al., 2016; Shan H. B. et al., 2016). The Motivation and Engagement Wheel posits that anxiety plays a crucial role in influencing learning engagement. Certain individuals tend to defer or abstain from learning activities due to unfavorable appraisals of anxiety and other negative emotions, leading to reduced learning engagement (Martin, 2007; Török et al., 2018). In summary, in line with current research, individuals characterized by high anxiety traits, who are predisposed to experiencing anxiety when confronted with academic tasks, may utilize procrastination as a maladaptive avoidance tactic to alleviate bodily distress, thereby manifesting academic procrastination behavior. Consequently, this study postulates the first hypothesis: Trait anxiety positively predicts academic procrastination behavior.

### 1.2 The mediating role of academic self-efficacy

Academic self-efficacy is defined as self-perceived capability in competently accomplishing academic tasks at a particular level (Ji and Zhao, 2021). Existing research consistently demonstrates an obvious negative relation between anxiety and academic self-efficacy (Yao et al., 2010; Lei, 2019). Notably, college students characterized by trait anxiety exhibit biases in their strategy utilization patterns (Pan et al., 2019), and those with elevated trait anxiety tend to employ specific strategies more frequently, such as expressive suppression and experiential avoidance (Zhang, 2011; O'Toole et al., 2017). Both inhibition and avoidance may lead to low levels of confidence in completing academic tasks, indicating low academic self-efficacy. Meanwhile, individuals characterized by elevated academic self-efficacy demonstrate a decreased likelihood of experiencing anxious emotions, such as test anxiety (Warshawski et al., 2018). According to self-consistency theory, academic self-efficacy functions as a crucial psychological mechanism influencing various aspects of learning. These aspects encompass the selection of learning behaviors, persistence, exertion of effort, emotional responses, and coping styles (Ding, 2022). Individuals characterized by low self-efficacy are more susceptible to experiencing academic burnout, and the adverse effects further exacerbate academic procrastination (Song and Luo, 2018). Previous research about college students has identified that academic self-efficacy mediates perfectionist personal traits and academic procrastination behavior (Luo and An, 2022). Building upon this analysis and grounded in self-consistency theory, this study postulates the second hypothesis: Academic self-efficacy functions as a mediator linking trait anxiety and academic procrastination behavior.

### 1.3 The regulatory role of autonomous motivation in learning

The self-determination theory classifies human motivation by autonomy level: amotivation, extrinsic, and intrinsic types (Deci and Ryan, 1985). The self-determination components within these motivation types increase sequentially across this continuum. Additionally, extrinsic motivation is further subdivided into four constructs: extrojected regulation, introjected regulation, identification regulation, and integrated regulation. Amotivation implies an absence of interest, identification, or concern for the task’s value. Extrojected regulation involves individuals acting solely to pursue external goals such as financial gain or job promotion, or to avoid external punishment. Introjected regulation encompasses actions to avoid guilt, anxiety, or to maintain self-respect. Identification regulation entails actions aligned with the recognition of the task’s value, contributing to a decrease in the perceived sense of control and an increase in self-determination. Integrated regulation describes individuals fully accepting external rules and willingly adhering to them, with these external rules becoming entirely internalized as the individual’s internal needs. Lastly, intrinsic motivation signifies that individuals act entirely out of love and interest in the task (Deci and Ryan, 2002). Canadian psychologist Vallerand et al. compiled the Academic Motivation Scale College Version (Vallerand et al., 1993), grounded in the Self-Determination Theory, which is widely employed for assessing learning motivation, with ongoing research in this area. Both national and international studies have indicated that motivation is markedly negatively correlated with academic self-efficacy. In contrast, extrinsic motivation exhibits no correlation with academic self-efficacy, while intrinsic motivation shows a statistically robust positive association with academic self-efficacy (Walker et al., 2006; Chi and Xin, 2006). It is evident that individuals with varying levels of autonomous learning motivations undergo distinct experiences regarding anxiety and academic self-efficacy. Consequently, the study raises the third hypothesis: For the impact of Autonomous Motivation in Learning on the pathway linking trait anxiety to academic procrastination, the initial segment of the mediating function involving academic self-efficacy is influenced by the moderating impact of academic self-determination motivation.

### 1.4 The current study

**Figure 1:**
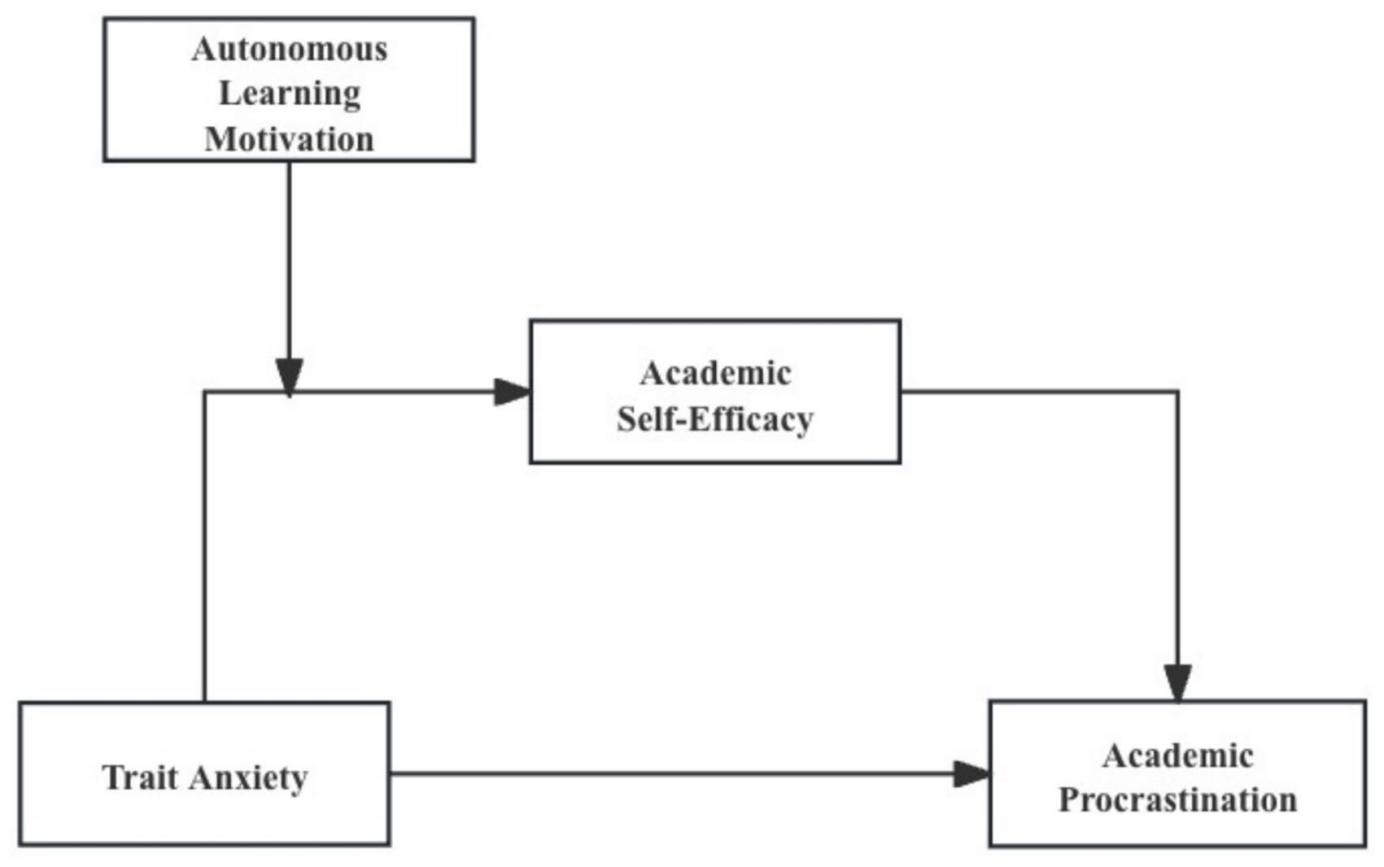
The moderated mediation model.

The above theoretical derivation suggests a moderated mediation model (Figure 1) was formulated to explain how trait anxiety affects academic procrastination among college students, in conjunction with the prospective mechanisms involving academic self-efficacy and autonomous motivation for learning, aiming to offer novel theoretical insights and effective interventions aimed at mitigating academic procrastination among students by identifying potential influencing factors.

## 2 Materials and methods

### 2.1 Research objectives

This survey was conducted online through Questionnaire Star. College students from different universities in Zhejiang Province were invited to fill it out in a quiet environment in their class units. Data collection yielded 3,832 authenticated responses. Exceptions were eliminated where the repetition rate of answer options exceeded 70% and the answer time was outside three standard deviations. The final dataset comprised 3,370 qualified responses, achieving a validity ratio of 87.94%. Participant age: M = 19.41, SD = 4.48. The distribution of research subjects on variables such as gender, whether they are an only child, grade, household registration location, etc., is presented below (Table 1).

**Table 1:** Statistical overview of students’ demographic information (*n* = 3,370).

Variable	Category	Amount	Percentage (%)
Gender	Male	2,216	65.76
	Female	1,154	34.24
From only child family	Yes	1712	50.8
	No	1,658	49.2
Grade	Freshman	1,398	41.48
	Sophomore	1,622	48.13
	Junior	280	8.31
	Senior	70	2.08
Distribution	Urban	1,468	43.56
	Town	622	18.46
	Rural	1,280	37.98

### 2.2 Measurement instruments

#### 2.2.1 Academic procrastination

The study employed the Irrational Procrastination Scale developed by Steel (2007), comprising 9 behavioral statements on a Likert scale from “1” denoting “I would never or rarely engage in this behavior” to “5” indicating “I always or consistently engage in this behavior.” Higher scores reflect greater levels of irrational procrastination. Scores are categorized as follows: total score ≤ 23 points signifies a low level; scores from 23 to 32 points correspond to a moderate level; and scores ≥ 32 points denote a high level of irrational procrastination (*α* = 0.725).

#### 2.2.2 Trait anxiety assessment

The Trait Anxiety Inventory from the State–Trait Anxiety Inventory, initially developed by Speilberger et al. (1983) and culturally adapted by Zheng and Li (1997), was utilized, comprising 20 items. The scale ranges from 1 (rarely) to 4 (almost always). High total scores on the scale indicate elevated levels of trait anxiety (*α* = 0.913).

#### 2.2.3 Academic self-efficacy

The Academic Self-Efficacy Scale was originally constructed by Liang (2000) from Central China Normal University, drawing inspiration from the research of Pintrich and DeGroot (1990). It comprises 22 items using a 1–5 consistency scale, where 1 indicates total inconsistency and 5 represents complete consistency. High scores indicate elevated academic self-efficacy (*α* = 0.914).

#### 2.2.4 Autonomous learning motivation

The autonomous learning motivation scale utilized in this study was compiled by Vallerand et al. (1992) and revised into the Chinese version by Chen (2007). Comprising 27 items, the scale quantifies the degree of self-determined motivation within the learning domain. High total scores on the scale reflect elevated an level of autonomous learning motivation (*α* = 0.901).

### 2.3 Statistical procedure

Data entry and processing were conducted using SPSS 21.0 and its plugin PROCESS. Statistical analyses comprising descriptive statistics, *t*-tests, ANOVA, correlation analysis, and regression analysis were employed to detect relevant variable associations, mediating effects, and moderating effects.

## 3 Results

### 3.1 Academic procrastination

#### 3.1.1 Overview

**Figure 2:**
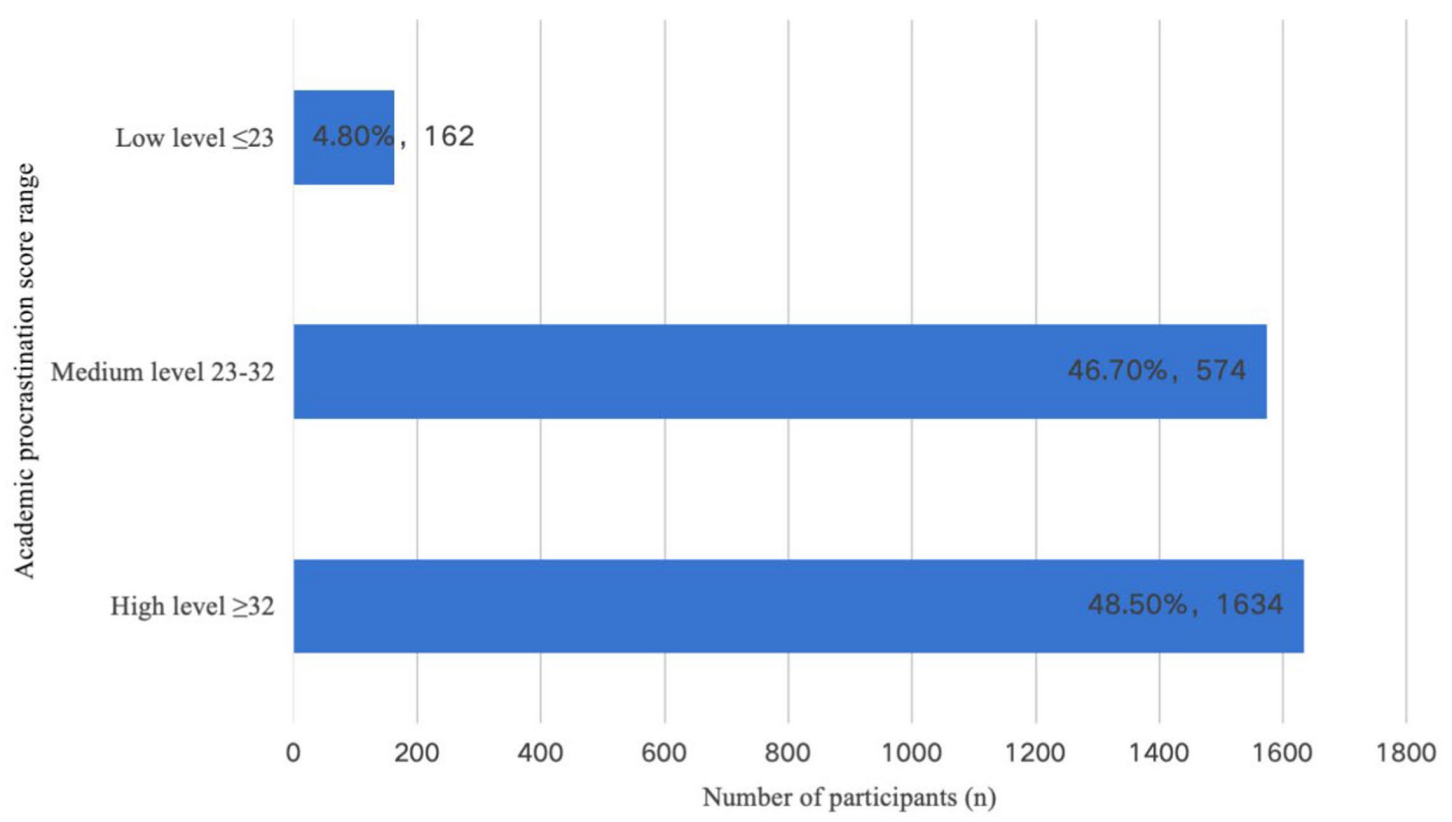
Distribution of participants with academic procrastination levels.

Among the 3,370 undergraduate participants in this survey, the manifestation of irrational academic procrastination behavior was generally indicative of a moderate level, with the mean academic procrastination score of 23.18 (SD = 5.33). The distribution of academic procrastination levels is shown in Figure 2. The graphical representation illustrates that over 50% of participants demonstrate moderate to high levels of irrational academic procrastination tendencies.

#### 3.1.2 Variations in academic procrastination

Statistical analyses were conducted in SPSS 26.0. The outputs are presented in Table 2.

**Table 2:** Association between academic procrastination total scores and different participants.

Variable	Family annual income (*r*)	Grade level (*r*)	Grade ranking percentage (*r*)
Academic procrastination total score	−0.049*	0.024	0.191**

* indicates *p* < 0.05, ** indicates *p* < 0.01, *** indicates *p* < 0.001 (two-tailed tests). Similarly for subsequent analyses.

The grade level of participants was found to be unrelated to their levels of academic procrastination. Family income demonstrated a significant negative correlation with academic procrastination levels, while grade ranking showed a significant positive correlation with total academic procrastination scores.

The independent sample *t*-tests indicated the gender comparison showed negligible effects on procrastination scores (t = −0.56, *p* = 0.57; male academic procrastination total score M = 23.13, SD = 5.30; female academic procrastination total score M = 23.28, SD = 5.40). Correspondingly, the comparison of family structure also demonstrated nonsignificant effects (t = −0.76, *p* = 0.45; only-child academic procrastination total score M = 23.09, SD = 5.30; non-only-child academic procrastination total score M = 23.28, SD = 5.37).

One-way analysis of variance (ANOVA) indicated no significant differences in academic procrastination total scores among undergraduates from different household registrations (*F* = 0.35, *p* = 0.70), varying family annual incomes (*F* = 1.12, *p* = 0.22), or different grade levels (*F* = 0.61, *p* = 0.61).

### 3.2 The overview of trait anxiety, academic self-efficacy, and autonomous learning motivation

#### 3.2.1 Trait anxiety

The mean trait anxiety level among participants was 42.43 (SD = 9.8).

An independent sample *t*-test was conducted in this study, and it showed significant differences in trait anxiety levels between genders (t = −3.8, *p* < 0.01), with females exhibiting higher trait anxiety levels (M = 43.68, SD = 9.91) compared to males (M = 41.78, SD = 9.69). Additionally, non-only-child participants demonstrated significantly higher levels of trait anxiety than only-child participants (t = −2.71, *p* < 0.01), with mean trait anxiety scores of 43.09 (SD = 9.39) and 41.80 (SD = 10.15), respectively.

#### 3.2.2 Academic self-efficacy

High total scores indicate elevated academic self-efficacy in this scale, with a maximum value of 110 and a minimum value of 22. Analysis of the survey data revealed a mean total score of 75.78 (SD = 12.02) among the 3,370 participants, suggesting a moderate degree of academic self-efficacy.

Independent sample *t*-tests indicated obvious differences across gender (t = 3.58, *p* < 0.01), with males reporting higher levels of academic self-efficacy (M = 76.53, SD = 11.89) compared to females (M = 74.33, SD = 12.12). Moreover, only-child participants exhibited significantly stronger academic self-efficacy compared to non-only-child peers (t = 2.42, *p* < 0.05), with mean scores of 76.48 (SD = 12.13) and 75.06 (SD = 11.86), respectively.

#### 3.2.3 Autonomous learning motivation

The Autonomous Learning Motivation Scale utilized in this study computes a weighted total score across various dimensions to serve as a relative index of autonomous learning motivation. Higher scores indicate stronger autonomous learning motivation. Analysis of the survey data revealed a mean autonomous learning motivation index of 59.37 (SD = 16.21) among the 3,370 participants, with the highest score reaching 112 and the lowest reaching −16.

Gender and only-child status variables did not significantly differ in self-directed learning motivation, according to independent sample *t*-tests (*p* > 0.05).

### 3.3 Examination of common method bias

Data were collected through questionnaire surveys in the current research, where respondents were relatively consistent in their responses, and the measurement environment and methods were uniform. This uniformity may increase the likelihood of errors and result in homogenized outcomes that fail to effectively differentiate results. Therefore, prior to conducting a correlation analysis, a preliminary examination of the single-factor method was employed.

Exploratory factor analysis in Table 3 is as follows.

**Table 3:** Explained total variances of trait anxiety, self-efficacy, autonomous learning motivation, and academic procrastination scales.

Component	Initial eigenvalue			Extracted sum of squares and loadings		
	Total	Variance (%)	Cumulative (%)	Total	Variance (%)	Cumulative (%)
1	20.738	26.25	26.25	20.738	26.25	26.25
2	8.418	10.655	36.905	8.418	10.655	36.905
3	4.851	6.141	43.046	4.851	6.141	43.046
4	3.574	4.525	47.57	3.574	4.525	47.57
5	2.665	3.373	50.943	2.665	3.373	50.943
6	2.262	2.863	53.806	2.262	2.863	53.806
7	2.142	2.711	56.518	2.142	2.711	56.518
8	1.543	1.953	58.471	1.543	1.953	58.471
9	1.48	1.874	60.345	1.48	1.874	60.345
10	1.078	1.365	61.71	1.078	1.365	61.71
11	1.039	1.315	63.025	1.039	1.315	63.025

### 3.4 Descriptive statistics and differential analysis of trait anxiety, academic procrastination, self-deterioration, and autonomous learning motivation

Table 4 presents a significant correlation among trait anxiety, academic self-efficacy, autonomous learning motivation, and academic procrastination (*p* < 0.001). Specifically, trait anxiety and academic procrastination exhibited a remarkable positive relation, while academic self-efficacy showed a strong positive association with autonomous learning motivation. Furthermore, academic self-efficacy is significantly negatively related to trait anxiety and academic procrastination, while autonomous learning motivation is significantly negatively correlated with trait anxiety and academic procrastination.

**Table 4:** The mean values, standard deviations, and correlation coefficients for each variable.

Variable	*M*	*SD*	Academic procrastination	Trait anxiety	Academic self-efficacy	Autonomous learning motivation
Academic procrastination	23.19	5.33				
Trait anxiety	42.44	9.8	0.53***			
Academic self-efficacy	75.77	12.02	−0.40***	−0.58***		
Autonomous learning motivation	59.35	16.21	−0.22***	−0.37***	0.54***	
Family annual income	24.48	20.2	−0.05*	−0.04	0.10***	0.06*
Grade	1.71	0.71	0.02	0.04	−0.05*	−0.05*
Grade ranking	40.69	27.5	0.19***	0.10***	−0.26***	−0.15***

* indicates *p* < 0.05; *** indicates *p* < 0.001.

### 3.5 Mediation analysis of academic self-efficacy

The method established by Wen et al. (2004) is utilized to investigate the potential mediating function of academic self-efficacy. The dependent variable was represented by academic procrastination, the independent variable by trait anxiety, and the mediating variable by academic self-efficacy. Following the standardization of data pertaining to these three variables, alongside controlling for the family’s annual income and performance ranking, the subsequent table is derived through regression analysis, as displayed in Table 5.

**Table 5:** Mediation analysis of academic self-efficacy between trait anxiety and academic procrastination (*n* = 3,370).

Variable	Academic self-efficacy		Academic procrastination			
	Model 7	Model 8	Model 9	Model 10	Model 11	Model 12
Constant	79.18	107.55	21.92	10.39	35.28	15.63
Control the method proposed by Wen et al. (2004) variables						
Family annual income	0.08**	0.06**	−0.03	−0.02	−0.004	−0.01
Grade ranking	−0.26***	−0.20***	0.19***	0.14***	0.09***	0.12***
Independent variable						
Trait anxiety		−0.56***		0.51***		0.45***
Mediating variable						
Academic self-efficacy					−0.38***	−0.11***
*F*	68.08***	347.76***	32.95***	235.40***	115.97***	183.21***
*R* ^2^	0.08	0.38	0.04	0.30	0.17	0.30
Δ*R*^2^	0.08	0.31	0.04	0.26	0.13	0.27

** indicates *p* < 0.01; *** indicates *p* < 0.001.

In Model 7 of Table 5, the annual family income, as part of the control variables, has a significantly positive impact on academic self-efficacy (*β* = 0.08, *p* < 0.01), while the grade ranking percentage markedly and negatively impacts academic self-efficacy (*β* = −0.26, *p* < 0.001). Model 8 reveals a significant negative association between trait anxiety and academic self-efficacy (*β* = −0.56, *p* < 0.001). Following the adjustment for other variables, Model 10 demonstrates a notable positive relation between trait anxiety and procrastination (*β* = 0.51, *p* < 0.001). Additionally, Model 11 indicates a substantial inverse correlation between academic self-efficacy and procrastination (*β* = −0.38, *p* < 0.001). Subsequently, Model 12 examines the intricate relationship between trait anxiety, academic self-efficacy, and academic procrastination. The analysis underscores that academic self-efficacy mediates between trait anxiety and procrastination (*β* = −0.11, *p* < 0.001).

Moreover, employing the Process plug-in developed by Hayes, this study systematically aligns the independent, dependent, and mediating variables, and estimates resampling-derived 95% CIs for indirect effects (*n* = 3,370). The interplay among academic self-efficacy, trait anxiety, and academic procrastination is examined, with the mediating effect findings delineated in Table 6.

**Table 6:** Mediation analysis of academic self-efficacy between trait anxiety and academic procrastination (*n* = 3,370).

Effect	Effect value	Boot standard error	Boot CI lower limit	Boot CI upper limit
Mediation effect of academic self-efficacy	0.168	0.023	0.123	0.215
Direct effect	0.342	0.030	0.284	0.401
Total effect	0.510	0.021	0.470	0.551

**Figure 3:**
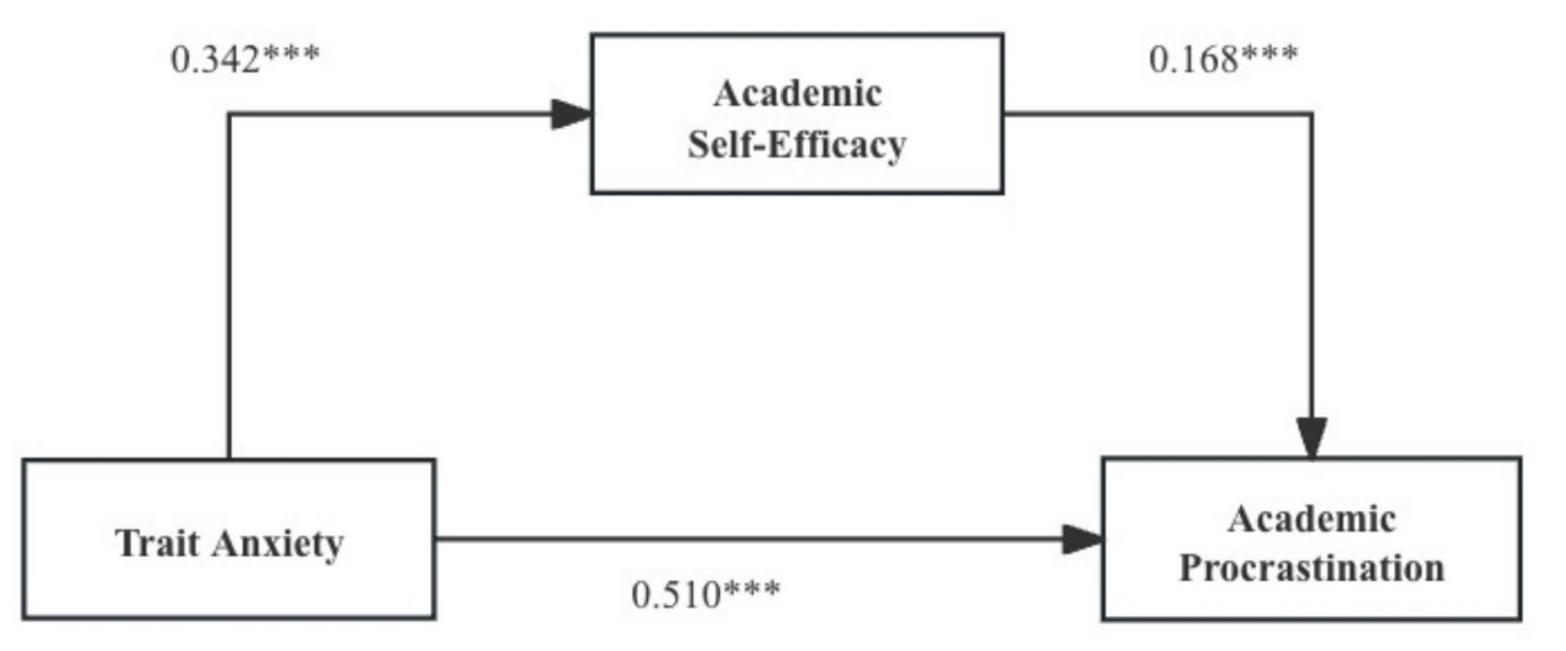
Schematic diagram illustrating the mediation process of academic self-efficacy.

As shown in Table 6, within the 95% confidence interval, academic self-efficacy exhibits a mediation function between trait anxiety and academic procrastination. This finding reinforces the outcomes derived from the regression analysis concerning the mediating role. The mediation process is presented in Figure 3.

### 3.6 Examination of the moderated mediation model

In accordance with the hypothesis, there can be a moderated mediation model when learning self-determination motivation plays a moderating role connecting the independent variable, trait anxiety and the mediating variable academic self-efficacy. This study employs Model 7 of the SPSS plug-in PROCESS, developed by Hayes for examination of the moderated mediation model. Trait anxiety serves as the independent variable, academic procrastination as the dependent, academic self-efficacy as the mediating, and learning self-determination motivation as the moderator. Following the control for family annual income and grade ranking, the statistical findings are presented in Tables 7, 8.

**Table 7:** Examination of the moderated mediation model.

Predictor variable	Academic self-efficacy			Academic procrastination		
	*β*	SE	t	*β*	SE	t
Family annual income	0.002	0.001	2.771**	−0.001	0.001	−0.483
Grade ranking	−0.006	0.001	−9.198***	0.004	0.001	5.544 ***
Trait anxiety	−0.043	0.019	−22.887***	0.448	0.025	17.863***
Academic self-efficacy				−0.113	0.026	−4.363***
Autonomous learning motivation	0.353	0.019	18.547***			
Trait anxiety × autonomous learning motivation	0.032	0.013	2.502*			
*R* ^2^		0.002			0.304	
*F*		6.258***			183.208***	

*indicates *p* < 0.05; **indicates *p* < 0.01; ***indicates *p* < 0.001.

**Table 8:** Examination of the moderating effect of autonomous learning motivation.

Effect	Effect value	Boot standard error	Boot CI lower limit	Boot CI upper limit
The moderating effect of autonomous learning motivation	−0.004	0.002	−0.008	0.000

**Figure 4:**
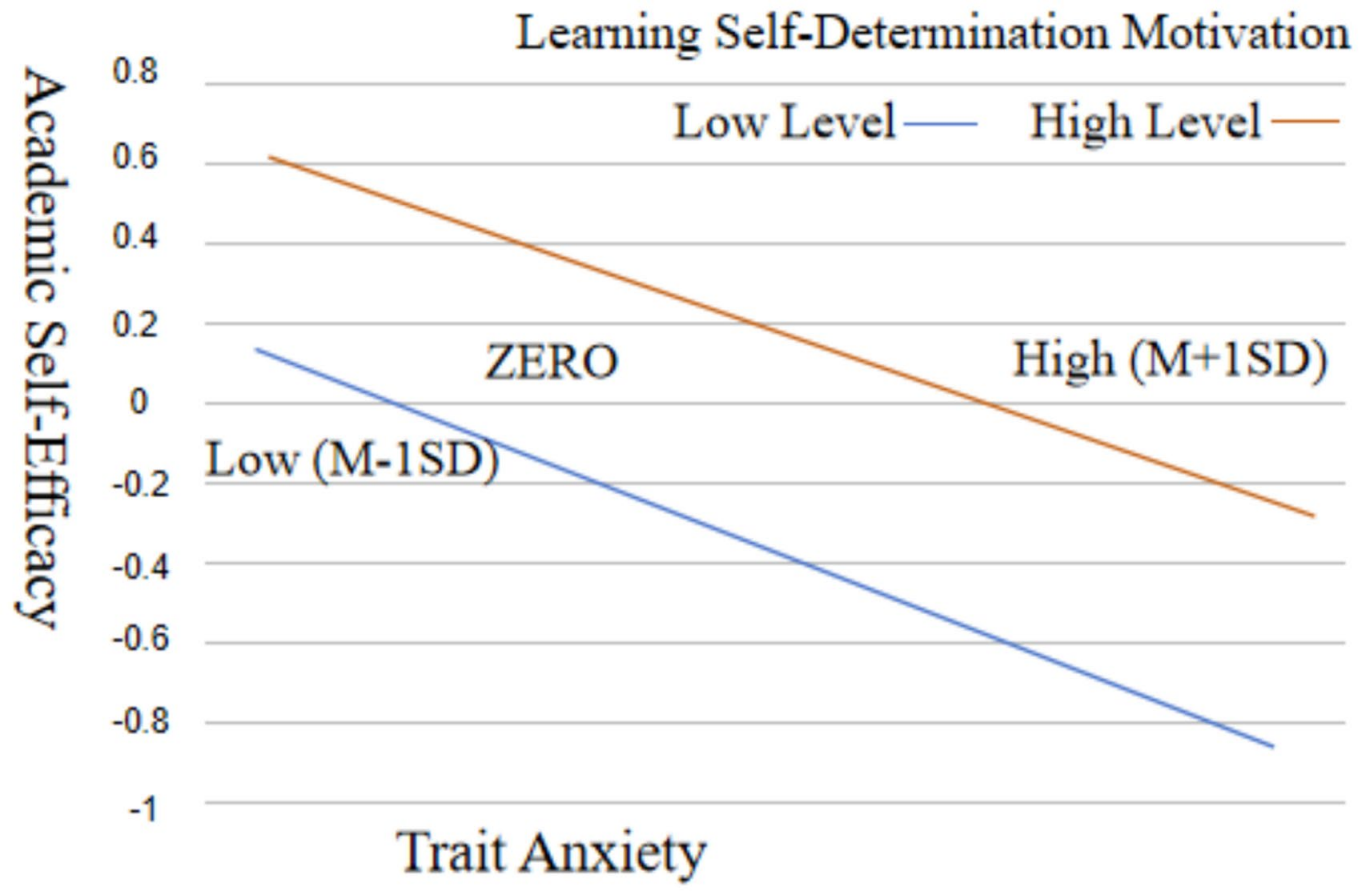
Moderating influence of learning self-determination motivation on the association between trait anxiety and academic self-efficacy.

Table 7 demonstrates that upon incorporating learning self-determination motivation into the model, trait anxiety significantly predicts academic self-efficacy (*β* = −0.043, *p* < 0.001). Furthermore, the interaction effect of trait anxiety × autonomous learning motivation on academic efficacy is statistically significant (*β* = 0.032, *p* < 0.001). These results show that learning self-determination motivation exerts a moderating influence between trait anxiety and academic self-efficacy. The moderating effect is −0.004, and the moderating impact of learning self-determination motivation is statistically significant. For further examination, the aggregate score of trait anxiety was stratified into high and low categories using the mean score plus or minus one standard deviation, facilitating a straightforward slope analysis. The ensuing outcomes are depicted in Figure 4.

Evidently, the depicted trend underscores that, particularly when the level of independent learning motivation is low, trait anxiety demonstrates a more pronounced effect on academic self-efficacy.

## 4 Discussion

Building upon the previous studies of academic procrastination and the prevalent characteristics of college students, this study empirically tested a moderated mediation model to delineate how trait anxiety affects academic procrastination via the mediating function of academic self-efficacy, with autonomous motivation in learning serving as a moderator. This can not only deepen our understanding of college students’ academic procrastination but also provide a certain theoretical basis for academic procrastination intervention.

### 4.1 Analysis of trait anxiety, academic self-efficacy, autonomous learning motivation, and academic procrastination, and demographic differences

This investigation reveals that college students’ academic procrastination tends to be at a moderate level, with over 50% exhibiting moderate or severe procrastination tendencies. Notably, no significant differences were observed in academic procrastination levels among students concerning gender, household registration location, major, or grade. Previous studies have also indicated inconclusive findings regarding gender disparities in academic procrastination, with its impact remaining unpredictable (Steel, 2007). Academic procrastination is pervasive among college students to a certain extent. The overall trait anxiety level among surveyed subjects averaged 42.43 (SD = 9.80). In terms of gender dimension, the female exhibited significantly higher levels of trait anxiety relative to the male, aligning with extant literature (Ji and Zhao, 2021). Physiological factors may contribute to the greater challenge the female faces in achieving equivalent accomplishments as the male, resulting in heightened stress levels and trait anxiety among female college students. Similarly, due to heightened peer competition, non-only children demonstrated higher trait anxiety levels compared to only children. The total academic self-efficacy score of the 3,370 subjects averaged 75.78 (SD = 12.02), positioning the moderate level of overall academic self-efficacy. Besides, the male scored higher than the female, potentially attributed to societal and familial expectations placing greater responsibilities and aspirations on men, thereby fostering a higher sense of self-efficacy among boys. Similarly, students from only children family exhibited higher academic self-efficacy scores compared to those from non-only children family. The mean value of the autonomous learning motivation index among college students was 59.37, with a standard deviation of 16.21, spanning from −16 to 112. Substantial variations were observed in the autonomous learning motivation among different college students, with no discernible differences based on gender or only-child status. Autonomous learning motivation is a multifaceted variable. The Chinese version of the Autonomous Learning Motivation Scale (Chen, 2007) utilizes a weighted total score for each dimension to compute the relative autonomous learning motivation index (autonomous learning motivation = 2 × intrinsic motivation + identification regulation − introjected regulation − 2 × external regulation). This index exhibits substantial variability among individuals and cannot be simply distinguished by demographic variables.

### 4.2 Relationship analysis between trait anxiety, academic self-efficacy, learning self-determination motivation, and academic procrastination

#### 4.2.1 Association between trait anxiety and academic procrastination

This study reveals a significant positive correlation between trait anxiety and academic procrastination. Further regression analysis indicates that trait anxiety positively predicts academic procrastination. Individuals with elevated trait anxiety easily have academic procrastination behavior, thereby validating hypothesis 1. This result aligns with previous research outcomes (Shan H. B. et al., 2016; Tang et al., 2015; Yue et al., 2022), which suggests anxiety can be a significant predictive factor for academic procrastination. As delineated in the anxiety activation model proposed by Spielberger (1966), individuals tend to engage in behaviors aimed at alleviating unpleasant emotions they experience. Particularly, individuals with elevated trait anxiety demonstrate increased susceptibility to encounter negative emotions like nervousness, depression, and tension. When dealing with challenging tasks or responsibilities, individuals characterized by high trait anxiety exhibit a propensity to procrastinate actively, as a strategy to circumvent the potential consequences associated with the task. This behavioral pattern allows them to temporarily mitigate feelings of stress and anxiety (Lu, 2011). Consequently, it can be posited that trait anxiety exerts a notable predictive influence on procrastination behavior and directly shapes individuals’ tendencies toward procrastination. This finding is corroborated with current findings (Ali, 2025) that neurotic symptoms of trait anxiety and the most prominent dimension of psychological vulnerability were positively correlated with academic procrastination, and psychological vulnerability could account for 66.3% of the variance in academic procrastination among university students. In addition, structural equation modeling (Özbay et al., 2025) further demonstrates that academic procrastination is shaped by multiple interacting psychological and digital-related variables.

#### 4.2.2 Mediating role of academic self-efficacy

Academic self-efficacy is revealed to have a mediating function between trait anxiety and academic procrastination. Previous research suggests that individuals tend to take action to reduce their anxiety and enhance self-efficacy when the goal is close and achievable, and the individual will develop a tendency toward motivation (Liu et al., 2018). Academic self-efficacy significantly influences students’ cognition, emotions, and behavior while learning. Learners with low self-efficacy tend to avoid challenges and difficulties, leading to negative emotions in learning and susceptibility to depression and anxiety when facing academic pressure (Zhang, 2021). Academic self-efficacy has dual influences on academic procrastination behavior, both through direct effects and via an interactive relationship with emotions such as anxiety arising from academic tasks. Individuals with high trait anxiety but strong academic self-efficacy can transform moderate anxiety into a driving force for action, thereby reducing academic procrastination behavior. Examining the mediating function of academic self-efficacy provides an integrative framework for how trait anxiety generates academic procrastination behavior. This mediating mechanism is further supported by Karagöz and Özbay (2025) who identified academic procrastination as a mediating variable linking nomophobia and netlessphobia among nursing students, highlighting its bridging function within digital anxiety frameworks.

#### 4.2.3 Moderating role of autonomous learning motivation

The current study also examined whether autonomous learning motivation moderates the first half path of “trait anxiety → academic self-efficacy → academic procrastination behavior” (hypothesis 3 was tested). The findings reveal that when individuals have lower levels of autonomous learning motivation, the negative predictive impact of trait anxiety on academic self-efficacy becomes stronger, resulting in elevated academic procrastination. The Autonomous Learning Motivation Scale with seven dimensions calculates the weighted total score of each dimension as the relative autonomous learning motivation index, representing individual tendency toward autonomous learning motivation (Chen, 2007). Self-determination theory posits that when motivation for a target behavior is either amotivation or controlled motivation, there is almost no sense of identification and efficacy for the target behavior (Deci and Ryan, 2002). According to the calculation method of the autonomous learning motivation index, the lower the individual’s motivation tends toward amotivation or controlled motivation, the lower the index. Thus, when autonomous learning motivation is low, those with elevated trait anxiety tend to have very low academic self-efficacy. Previous research suggests that individuals with high self-efficacy possess confidence in completing learning tasks promptly, exhibit stronger autonomy in completing learning tasks, and experience less academic procrastination behavior (Ozkal, 2019). Therefore, college students characterized by high trait anxiety and low autonomous learning motivation should be focused on intervening promptly and reducing the occurrence of academic procrastination behavior.

### 4.3 Educational implications

The findings of this study provide important implications for addressing academic procrastination among college students. First, educators should recognize that academic procrastination is not merely a manifestation of laziness or lack of motivation and that may be closely associated with students’ personality traits and psychological factors. In particular, the present study confirmed that trait anxiety influences academic procrastination through academic self-efficacy and autonomous learning motivation. This mechanism offers an important reference for intervention. Therefore, interventions targeting academic procrastination should take into account students’ mental health status as well as motivational factors such as academic self-efficacy and autonomous learning motivation. As Demir and Kuşcu Karatepe (2025) found that academic procrastination exerts a significant negative effect on the life satisfaction of nursing and midwifery students, with academic self-efficacy and self-control serving as key serial mediators, and enhancing these two psychological resources can effectively mitigate the aforementioned negative impact. Educators may guide students to set phased goals, establish detailed feedback mechanisms, promote positive attribution styles, and cultivate sustained experiences of achievement. These strategies can help students build self-confidence and reduce the occurrence of academic procrastination.

Second, universities should strengthen mental health education. Special attention should be given to helping students develop adaptive coping strategies. A series of structured activities may be implemented to enhance academic self-efficacy and autonomous learning motivation. For example, career planning guidance, physical exercise programs, relaxation training, and time management training can help alleviate anxiety and stress because these measures may assist students in establishing clear learning goals and plans, improving self-control, and increasing learning efficiency.

Finally, students themselves need to understand and analyze the underlying causes and mechanisms of their academic procrastination. On the one hand, they should enhance autonomy and self-regulation, improve time management skills, practice self-acceptance, and cultivate positive emotions in order to cope effectively with academic pressure. On the other hand, students should reflect on their prior experiences of success and examine both individual and environmental factors contributing to procrastination, such as insufficient self-efficacy, low autonomous motivation, and anxiety-related constraints. By setting clear learning goals and implementing incremental incentive mechanisms, students can build a supportive environment for self-acceptance and personal growth. Through goal clarification and proactive engagement, they may continuously enhance academic self-efficacy and autonomous learning motivation, gradually break the vicious cycle between anxiety and procrastination, and ultimately escape the procrastination cycle.

## 5 Conclusion

The study utilized a relatively extensive sample to investigate the inquiry, “What are the predictive relationships between anxiety traits and procrastination patterns in undergraduate cohorts?.” This study contributes novel empirical evidence regarding the factors underlying academic procrastination among students and proposes effective intervention strategies. Specifically, the findings indicate that trait anxiety significantly and positively predicts college students’ engagement in academic procrastination behavior. Furthermore, academic self-efficacy partially mediates trait anxiety and academic procrastination. Moreover, the initial segment of the mediating effect of academic self-efficacy is moderated by autonomous motivation for learning, with trait anxiety exhibiting a more pronounced impact on academic procrastination among individuals with a low autonomous motivation index for learning. These outcomes underscore the importance of prioritizing attention to student cohorts characterized by high trait anxiety to facilitate a nuanced understanding of anxiety and foster healthy development among college students.

## Data Availability

The data analyzed in this study is subject to the following licenses/restrictions: For the data involved in this study, to protect students’ privacy, the school does not permit public disclosure of the data. If needed, the data can be requested from the corresponding author. Requests to access these datasets should be directed to 17376532661@163.com.

## References

[ref1] Ali A. (2025). Psychological-academic challenges: the relationship between psychological vulnerability and academic procrastination among university students. J. Palestine Ahliya Univ. Res. Stud. 4, 105–118. doi: 10.59994/pau.2025.3.105

[ref2] Chen B. H. (2007) *A preliminary investigation of academic procrastination in college students.* (master's thesis). East China Normal University, Shanghai, China

[ref3] Chi L. P. Xin Z. Q. (2006). The measure of learning motivation and the relationship between it and self-efficacy of college students. Psychol. Dev. Educ. 2, 64–70. doi: 10.3969/j.issn.1001-4918.2006.02.012

[ref4] Conti R. (2000). College goals: do self-determined and carefully considered goals predict intrinsic motivation, academic performance, and adjustment during the first semester. Soc. Psychol. Educ. 4, 189–211. doi: 10.1023/a:1009607907509

[ref5] Deci E. L. Ryan R. M. (1985). The general causality orientations scale: self-determination in personality. J. Res. Pers. 19, 109–134. doi: 10.1016/0092-6566(85)90023-6

[ref6] Deci E. L. Ryan R. M. (2002). Handbook of Self-Determination Research. 3rd Edn New York: University Rochester Press.

[ref7] Demir S. Kuşcu Karatepe H. (2025). The effect of academic procrastination on life satisfaction among nursing and midwifery students: the serial mediation role of academic self-efficacy and self-control. Behav. Sci. 15:1434. doi: 10.3390/bs15111434, 41301238 PMC12649465

[ref8] Ding C. L. (2022). The effect of professional commitment on academic procrastination: the mechanism of learning self-efficacy and learning motivation. J. West Anhui Univ. 38, 116–120+130. doi: 10.3969/j.issn.1009-9735.2022.02.023

[ref9] Gadosey C. K. Schnettler T. Scheunemann A. Fries S. Grunschel C. (2021). The intraindividual co-occurrence of anxiety and hope in procrastination episodes during exam preparations: an experience sampling study. Learn. Individ. Differ. 88:102013. doi: 10.1016/j.lindif.2021.102013

[ref10] Geng J. Y. Hou X. Yang H. Y. Han P. G. Gao F. Q. Han L. (2019). The relationship between trait anxiety and procrastination of college students: a moderated mediating effect. Stud. Psychol. Behav. 17, 402–407. doi: 10.3969/j.issn.1672-0628.2019.03.016

[ref11] Goroshit M. (2018). Academic procrastination and academic performance: an initial basis for intervention. J. Prev. Interv. Community 46, 131–142. doi: 10.1080/10852352.2016.1198157 29485387

[ref12] He N. (2019). The influence of trait anxiety and state anxiety on procrastination behavior. (master's thesis). Shanxi Normal University, Linfen, China

[ref13] Ji D. (2016). *The relationship between academic procrastination and self-control among college students*. (master’s thesis). Suzhou University, Suzhou, China

[ref14] Ji C. M. Zhao H. (2021). The relationship between teacher support, academic self-efficacy, and academic achievement of primary and middle school students: a meta-analytic structural equation model. Teach. Educ. Res. 33, 106–113. doi: 10.13445/j.cnki.t.e.r.2021.06.008

[ref15] Karagöz K. Özbay S. Ç. (2025). The mediating effect of academic procrastination on the relationship between nomophobia and netlessphobia in nursing students. Yükseköğretim Dergisi, 15, 281–290. doi: 10.53478/yuksekogretim.1522954

[ref16] Lei J. P. (2019). A correlational study of high school students' academic self-efficacy and test anxiety. Mental Health Educ. Primary Secondary Sch. 32, 21–23. doi: 10.3969/j.issn.1671-2684.2019.32.005

[ref17] Li Q. (2021). *The influence of future orientation and self-control on academic procrastination among high school students: intervention study*. (master’s thesis). Jilin Normal University, Jilin, China

[ref18] Li Y. H. Huo Z. Z. Wang X. K. Zhang L. B. Feng T. Y. (2021). Development of the academic procrastination scale for primary school students. Chin. J. Clin. Psychol. 29, 931–936. doi: 10.16128/j.cnki.1005-3611.2021.05.008

[ref19] Li S. R. Li J. Liu X. Q. (2016). Relationship between college students ' achievement motivation, anxiety and procrastination. Chin. J. Health Psychol. 24, 252–255. doi: 10.13342/j.cnki.cjhp.2016.02.025

[ref20] Li X. Lü H. (2022). The relationship between adolescents’ time attitudes and academic procrastination: the mediating role of achievement motivation. Psychol. Sci. 45, 47–53. doi: 10.16719/j.cnki.1671-6981.20220107

[ref21] Li F. Yu Y. Sun H. (2023). The effect of adolescent internet addiction on academic procrastination: a moderated mediation model. J. Taishan Univ. 45, 107–114.

[ref22] Liang Y. S. (2000) *Study on achievement goals, attribution styles and academic self-efficacy of college students*. (master's thesis). Central China Normal University, Wuhan, China

[ref23] Liu Y. Z. Yang Z. Y. Wang Y. Q. Chen J. Cai H. (2018). The concept of future self-continuity and its effects. Adv. Psychol. Sci. 26, 2161–2169. doi: 10.3724/SP.J.1042.2018.02161

[ref24] Liu J. Zhan T. Q. Jiang X. S. (2023). Study on the current situation of college students' academic procrastination and its cognitive behavior intervention. Psychol. Mon. 18, 91–93. doi: 10.19738/j.cnki.psy.2023.15.025

[ref25] Lu K. (2011). College students' self-efficacy, trait anxiety and coping styles related research. Soc. Psychol. Sci. Z1, 235–239.

[ref26] Luo J. An L. N. (2022). The influence of perfectionism on college students' academic procrastination: the mediating role of academic self-efficacy. Chin. J. Health Psychol. 38, 77–84. doi: 10.13391/j.cnki.issn.1674-7798.2022.11.004

[ref27] Martin A. J. (2007). Examining a multidimensional model of student motivation and engagement using a construct validation approach. Br. J. Educ. Psychol. 77, 413–440. doi: 10.1348/000709906X118036, 17504555

[ref28] O'Toole M. S. Zachariae R. Mennin D. S. (2017). Social anxiety and emotion regulation flexibility: considering emotion intensity and type as contextual factors. Anxiety Stress Coping 30, 716–724. doi: 10.1080/10615806.2017.1346792 28662586

[ref29] Özbay Ö. Doğan U. Adıgüzel O. Cinar Özbay S. (2025). Modeling factors associated with academic procrastination in university students. Psychol. Rep. 133:00332941251335573. doi: 10.1177/00332941251335573 40232064

[ref30] Ozkal N. (2019). Relationships between self-efficacy beliefs, engagement, and academic performance in math lessons. Cypriot J. Educ. Sci. 14, 190–200. doi: 10.18844/cjes.v14i2.3766

[ref31] Pan D. N. Wang Y. Li X. B. (2019). Strategy bias in the emotion regulation of high trait anxiety individuals: An investigation of underlying neural signatures using ERPs. Neuropsychology 33, 111–122. doi: 10.1037/neu0000471, 30614720

[ref32] Peixoto E. M. Pallini A. C. Vallerand R. J. Rahimi S. Silva M. V. (2021). The role of passion for studies on academic procrastination and mental health during the covid-19 pandemic. Soc. Psychol. Educ. 24, 877–893. doi: 10.1007/s11218-021-09636-9, 34121913 PMC8184402

[ref33] Pintrich P. R. DeGroot E. V. (1990). Motivational and self-regulated learning components of classroom academic performance. J. Educ. Psychol. 82, 33–40. doi: 10.1037/0022-0663.82.1.33

[ref34] Procee R. Kamphorst B. A. van Wissen A. Meyer J. C. (2013). A formal model of procrastination. In The Proceedings of the 25th Benelux Conference on Artificial Intelligence (BNAIC 2013) (pp. 152–159).

[ref35] Shan H. Zhang L. Wei M. . (2016). The mediating role of self-control between procrastination and anxiety among college students. Chin. J. Ment. Health 30, 624–628.

[ref36] Shan H. B. Zhang L. Y. Wei M. Xin Y. J. Quan S. A. Li Y. (2016). Mediating effect of self-control on relationship between procrastination and anxiety in college students. Chin. Ment. Health J. 30, 624–628. doi: 10.3969/j.issn.1000-6729.2016.08.012

[ref37] Sirois F. Pychyl T. (2013). Procrastination and the priority of short-term mood regulation: consequences for future self. Soc. Personal. Psychol. Compass 7, 115–127. doi: 10.1111/spc3.12011

[ref38] Song Y. Q. Luo Z. R. (2018). The relationships between learning burnout and education achievement attribution, academic self-efficacy of college students. Chin. J. Health Psychol. 26, 124–127. doi: 10.13342/j.cnki.cjhp.2018.01.033

[ref39] Song M. Su T. Feng T. (2015). A time-orientation model of procrastination behavior. Adv. Psychol. Sci. 23, 1216–1225. doi: 10.3724/SP.J.1042.2015.01216

[ref40] Speilberger C. D. Gorsuch R. Lushene R. . (1983). Manual for the state-trait anxiety inventory. Palo Alto, CA: Consulting Psychologists.

[ref41] Spielberger C. D. (1966). Theory and research on anxiety. Anxiety Behav. 1, 413–428. doi: 10.1016/B978-1-4832-3131-0.50006-8

[ref42] Steel P. (2007). The nature of procrastination: a meta-analytic and theoretical review of quintessential self-regulatory failure. Psychol. Bull. 133, 65–94. doi: 10.1037/0033-2909.133.1.65, 17201571

[ref43] Steel P. Taras D. Ponak A. Kammeyer-Mueller J. (2022). Self-regulation of slippery deadlines: the role of procrastination in work performance. Front. Psychol. 12:783789. doi: 10.3389/fpsyg.2021.783789, 35069365 PMC8770981

[ref44] Tang K. Q. Fan F. L. Ke C. S. J. Peng T. Yang Y. C. Peng T. (2015). Early maladaptive schemas, anxiety and procrastination among Chinese students. Psychol. Dev. Educ. 31, 360–736. doi: 10.16187/j.cnki.issn1001-4918.2015.03.14

[ref45] Török L. Szabó Z. P. Tóth L. A. (2018). Critical review of the literature on academic self-handicapping: theory, manifestations, prevention and measurement. Soc. Psychol. Educ. 21, 1175–1202. doi: 10.1007/s11218-018-9460-z

[ref46] Vallerand R. J. Pelletier L. G. Blais M. R. . (1992). The academic motivation scale: a measure of intrinsic, extrinsic, and amotivation in education. Educ. Psychol. Meas. 52, 1003–1017. doi: 10.1177/001316449205200402

[ref47] Vallerand R. J. Pelletier L. G. Blais M. R. Briere N. M. Senecal C. Vallieres E. F. (1993). On the assessment of intrinsic, extrinsic, and amotivation in education: evidence on the concurrent and construct validity of the academic motivation scale. Educ. Psychol. Meas. 53, 159–172. doi: 10.1177/0013164493053001018

[ref48] Walker C. O. Greene B. A. Mansell R. A. (2006). Identification with academics, intrinsic/extrinsic motivation, and self-efficacy as predictors of cognitive engagement. Learn. Individ. Differ. 16, 1–12. doi: 10.1016/j.lindif.2005.06.004

[ref49] Wang L. Gao Y. (2021). Achievement goal orientation and academic procrastination among graduate students in research universities: the mediating role of academic self-efficacy. Grad. Educ. Res. 63, 26–34. doi: 10.19834/j.cnki.yjsjy2011.2021.03.05

[ref50] Warshawski S. Bar-Lev O. Barnoy S. (2018). Role of academic self-efficacy and social support on nursing students' test anxiety. Nurse Eductor. 44, E6–E10. doi: 10.1097/NNE.0000000000000552, 29847355

[ref51] Wen Z. L. Zhang L. Hou J. T. Hau K. T. Liu H. (2004). Testing and application of the mediating effects. Acta Psychol. Sin. 36, 614–620. doi: 10.16128/j.cnki.1005-3611.2025.02.012

[ref52] Wu M. Zhang J. Ling D. Ye P. Q Deng Y. (2025). Is procrastination a vicious circle? The complex dynamic relationship between procrastination and anxiety among college students. Chin. J. Clin. Psychol. 33, 293–298.

[ref53] Xue L. (2024). *The impact of adolescent internet addiction, self-control, and anxiety on academic procrastination*. (master’s thesis). Chengdu Medical College, Chengdu, China

[ref54] Yang Z. Gao J. Ma J. Jiao J. L. (2025). The effect of academic procrastination on college students’ mobile phone dependence: chain mediation of cognitive emotion regulation strategies and anxiety. Sichuan Ment. Health 38, 59–64. doi: 10.11886/scjsws20240509004

[ref55] Yao J. Liu X. Li B. (2010). The impact of junior high school achievement goals and academic self-efficacy on test anxiety. J. Inner Mongolia Norm. Univ. (Educ. Sci. Edition) 23, 53–56. doi: 10.3969/j.issn.1671-0916.2010.06.017

[ref56] Yue P. F. Hu W. L. Zhang J. X. . (2022). Harsh parenting and learning engagement among middle school students: the role of state anxiety and gender. Psychol. Dev. Educ. 20, 226–232. doi: 10.12139/j.1672-0628.2022.02.012

[ref57] Zeng J. Li Y. (2019). Empirical research on academic procrastination of college students from the perspective of self-management. J. Tonghua Norm. Univ. 40, 132–138. doi: 10.13877/j.cnki.cn22-1284.2019.07.023

[ref58] Zhang S. J. (2011). *The impact of cognitive reappraisal and expressive suppression on emotional responses of college students with trait anxiety and training research.* (master's thesis). University of Electronic Science and Technology, Chengdu, China

[ref59] Zhang Y. X. (2021) *The relationship between trait anxiety and academic procrastination among college students.* (master's thesis). Hebei University, Baoding, China

[ref60] Zheng X. H. Li Y. Z. (1997). State-trait anxiety inventory. Chin. Ment. Health J. 4, 28–29.

